# Pneumothorax in Mechanically Ventilated Patients with COVID-19 Infection

**DOI:** 10.1155/2021/6657533

**Published:** 2021-01-11

**Authors:** Raziye Ecem Akdogan, Turab Mohammed, Asma Syeda, Nasheena Jiwa, Omar Ibrahim, Rahul Mutneja

**Affiliations:** ^1^Department of Internal Medicine, University of Connecticut, Farmington, Connecticut, USA; ^2^Department of Pulmonary and Critical Care Medicine, University of Connecticut, Farmington, Connecticut, USA; ^3^Department of Pulmonary and Critical Care Medicine, Saint Francis Hospital and Medical Center, Hartford, Connecticut, USA

## Abstract

Data on patient-related factors associated with pneumothorax among critically ill patients with COVID-19 pneumonia is limited. Reports of spontaneous pneumothorax in patients with coronavirus disease 2019 (COVID-19) suggest that the COVID-19 infection could itself cause pneumothorax in addition to the ventilator-induced trauma among mechanically ventilated patients. Here, we report a case series of five mechanically ventilated patients with COVID-19 infection who developed pneumothorax. Consecutive cases of intubated patients in the intensive care unit with the diagnosis of COVID-19 pneumonia and pneumothorax were included. Data on their demographics, preexisting risk factors, laboratory workup, imaging findings, treatment, and survival were collected retrospectively between March and July 2020. Four out of five patients (4/5; 80%) had a bilateral pneumothorax, while one had a unilateral pneumothorax. Of the four patients with bilateral pneumothorax, three (3/4; 75%) had secondary bacterial pneumonia, two had pneumomediastinum and massive subcutaneous emphysema, and one of these two had an additional pneumoperitoneum. A surgical chest tube or pigtail catheter was placed for the management of pneumothorax. Three out of five patients with pneumothorax died (3/5; 60%), and all of them had bilateral involvement. The data from these cases suggest that pneumothorax is a potentially fatal complication of COVID-19 infection. Large prospective studies are needed to study the incidence of pneumothorax and its sequelae in patients with COVID-19 infection.

## 1. Introduction

Pneumothorax, defined by the presence of air in the pleural cavity with or without collapse of the lung, is often a life-threatening complication and a medical emergency [1]. It can be classified into spontaneous, iatrogenic, or traumatic pneumothorax based on the etiology [2]. Iatrogenic pneumothorax occurs from a complication of a diagnostic or therapeutic intervention such as transthoracic-needle aspiration, placement of a central venous catheter, thoracentesis, lung, and or pleural biopsy, or barotrauma [3, 4]. During the coronavirus disease 2019 (COVID-19) pandemic, an increase in pneumothorax incidence, especially among mechanically ventilated patients with COVID-19 infection, has been observed. The mechanical ventilation has deleterious effects on the lung, and pneumothorax is a known complication of lung ventilation [5]. Ventilation-related pneumothorax has been more commonly reported in the pediatric population due to immature lung mechanics [6, 7]. Although spontaneous pneumothorax has been reported with infections, including COVID-19 [8, 9], the probability of pneumothorax increases from the combination of parenchymal injury from underlying infection and inflammatory response with additional positive pressure ventilation. A study on 202 patients from Wuhan, 12 (5.9%) patients developed pneumothorax on mechanical ventilation [10]. Here, we present a case series of five patients with pneumothorax among 150 patients of COVID-19 pneumonia admitted to the intensive care unit at a tertiary care center.

Information is summarized in Table 1.

## 2. Case Description

### 2.1. Case 1

A 62-year-old morbidly obese African American male patient with a past medical history of coronary artery disease (CAD) and obstructive sleep apnea (OSA) presented with seven days of progressive dyspnea. On presentation, he was hypoxic, and blood work was notable for leukocytosis. Chest X-ray (CXR) showed patchy bilateral opacities, and the COVID-19 polymerase chain reaction (PCR) test was positive. He was admitted for acute hypoxic respiratory failure secondary to COVID-19 pneumonia, which was further complicated with superimposed *Staphylococcus aureus* bacterial pneumonia. The patient's respiratory status deteriorated, and he was subsequently intubated on pressure control settings. He had a prolonged hospital course, and his treatment regimen included vasopressors, steroids, broad-spectrum antibiotic therapy, and convalescent plasma therapy without much clinical improvement. The following CXR demonstrated extensive subcutaneous emphysema.

**Figure 1:**
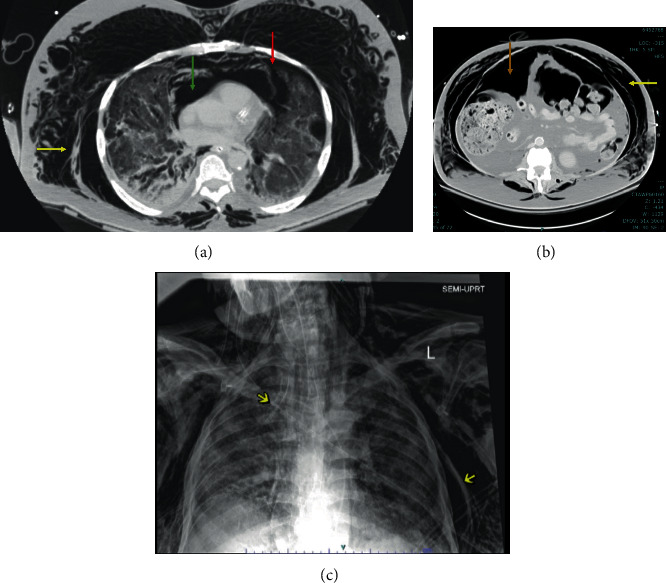
(a) CT of the chest showing moderate-sized left pneumothorax (red arrow), pneumomediastinum (green arrow), and extensive subcutaneous emphysema of the chest wall (yellow arrow). (b) Abdominal CT with large pneumoperitoneum (brown arrow) and subcutaneous emphysema of the abdominal wall (yellow arrow). (c) CXR demonstrating right-sided chest tube and subcutaneous emphysema of chest wall.

A computed tomography (CT) scan of the chest, abdomen, and pelvis revealed moderate bilateral pneumothoraces with pneumomediastinum and a large pneumoperitoneum (Figures 1(a) and 1(b)). Ventilation settings around the time of pneumothorax were pressure control/assist control mode (PC/AC), respiratory rate (RR) of 30 breaths per minute, inspiratory pressure (IP) 34 mmH20, inspiratory time (IT) 0.8 sec, positive end-expiratory pressure (PEEP) 10 cmH20, and the fraction of inspired oxygen (FiO2) 65%. Tube thoracostomy with a 28 French (Fr) surgical catheter was performed (Figure 1(c)). He had been intubated for a total of 49 days owing to the family values. However, given the patient's poor overall prognosis, his care was transitioned to comfort measures only.

### 2.2. Case 2

**Figure 2:**
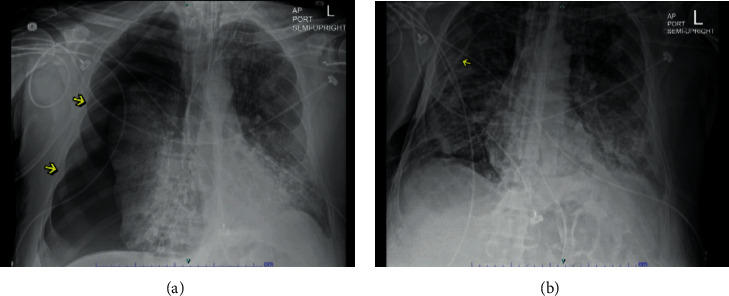
(a) CXR showing a large right-sided pneumothorax. (b) CXR showing resolution of the pneumothorax post chest tube placement.

A 60-year-old morbidly obese, Caucasian male patient with a past medical history of hypertension, presented with fever and nonproductive cough for three days. He was admitted for acute hypoxic respiratory failure secondary to COVID-19 pneumonia. A chest CT scan demonstrated extensive bilateral ground-glass opacities. The patient was intubated on pressure control settings due to a decline in respiratory status. His hospital course was further complicated by *Escherichia coli* pneumonia. He was treated with Remdesivir, broad-spectrum antibiotics, steroids, vasopressors, and convalescent plasma. The patient initially developed a small right-sided apical pneumothorax during the hospital course, which resolved spontaneously without intervention. However, a repeat CXR obtained a few days later revealed a large right-sided tension pneumothorax (Figure 2(a)), requiring immediate 24 Fr surgical chest tube placement (Figure 2(b)), further complicated by an air leak. At that time, the ventilation settings were ventilatory mode pressure regulated volume control (PRVC/AC), RR 26, tidal volume (TV) 650 ml, IP 28 cmH20, IT 1.1 second, PEEP 8 cmH20, and FiO2 80%. Subsequently, the patient also developed a left-sided pneumothorax requiring a 28 Fr surgical chest tube. Due to worsening bilateral pneumothoraces and increasing FiO2 requirements without clinical improvement, the patient's care was transitioned to comfort measures only.

### 2.3. Case 3

A 58-year-old Caucasian overweight male, a former smoker and hypertensive with no underlying lung disease, was admitted with acute hypoxic respiratory failure. Over the next few days, he deteriorated and was intubated for persistent hypoxemia. He received azithromycin and hydroxychloroquine, two doses of tocilizumab, and convalescent plasma therapy. He had been on pressure control (PC), and on day 16 of intubation, he underwent a bronchoscopy for evaluation of hemoptysis, which demonstrated bloody bronchoalveolar lavage. The next day he developed bilateral pneumothorax. He was placed on a 14 Fr pigtail catheter for left-sided pneumothorax and 28 Fr surgical chest tube for the right-sided tension pneumothorax. His ventilation settings were PC/AC, RR 22, PEEP of 8 cm H20, and FiO2 50%.

He underwent tracheostomy placement on day 21. Subsequently, the chest tubes were removed, and he was transitioned to a trach mask. The patient was doing well and undergoing chest physical therapy when he became acutely short of breath and hypotensive.

**Figure 3:**
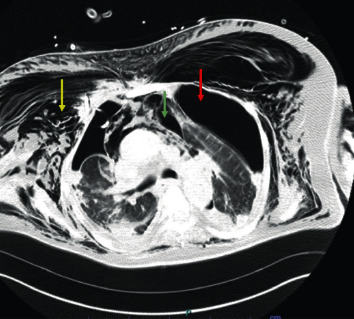
CT scan of the chest showing left-sided tension pneumothorax (red arrow), right-sided pneumothorax, pneumomediastinum (green arrow), and extensive subcutaneous emphysema of the chest wall (yellow arrow) with an indwelling right-sided chest tube.

A chest CT scan revealed a new right-sided pneumothorax, left-sided tension pneumothorax, pneumomediastinum, and subcutaneous emphysema of the chest wall (Figure 3). Emergent surgical chest tubes, 28 Fr on the right and 32 on the left, were placed again, and the patient was reintubated. He subsequently developed empyema and sepsis and eventually passed away.

### 2.4. Case 4

**Figure 4:**
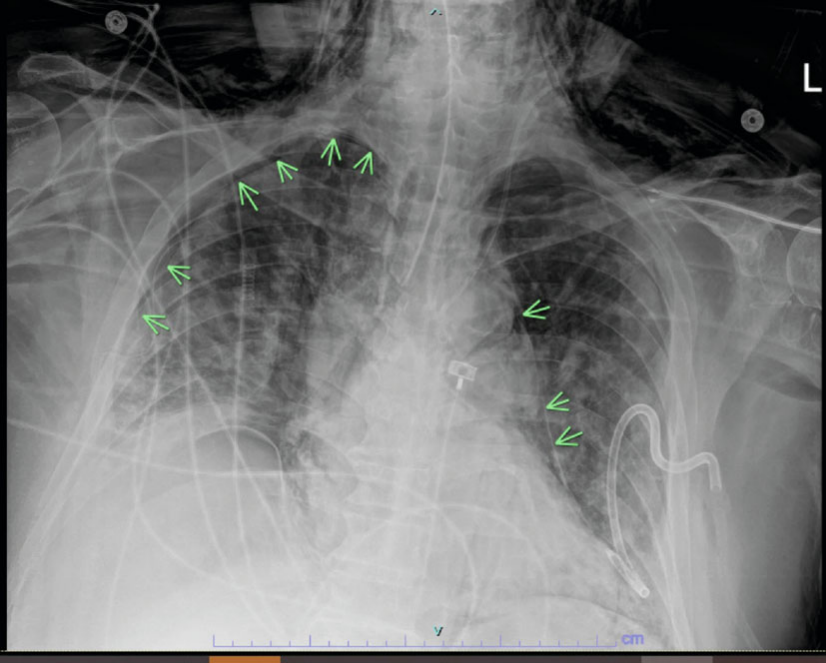
CXR showing persistent right-sided pneumothorax, pneumomediastinum, and the supraclavicular diffuse subcutaneous emphysema with a bilateral chest tube.

A 64-year-old morbidly obese Caucasian male with a past medical history of coronary artery disease presented with five days of fever and progressive shortness of breath. He had a 10-pack year smoking history; however, he had quit 40 years ago. He was hypoxic on admission and required high flow nasal cannula. He was subsequently intubated due to worsening hypoxia and was placed on pressure control settings. He developed superimposed *Staphylococcus aureus* and gram-negative bacterial pneumonia requiring multiple rounds of broad-spectrum antibiotics. He underwent tracheostomy and percutaneous endoscopic gastrostomy (PEG) tube placement. Later in his hospital course, his oxygen saturation dropped with a cuff leak from the tracheostomy tube, which prompted reintubation. The following CXR revealed bilateral pneumothoraces, and at that time, the ventilator settings were volume control/assist control mode (VC/AC), RR 20, TV 500 ml, PEEP 6 cmH2O, and FiO2 40%. He required bilateral tube thoracostomy with 28 Fr surgical chest tubes (Figure 4). He had a prolonged hospital course and was eventually discharged upon clinical improvement.

### 2.5. Case 5

**Figure 5:**
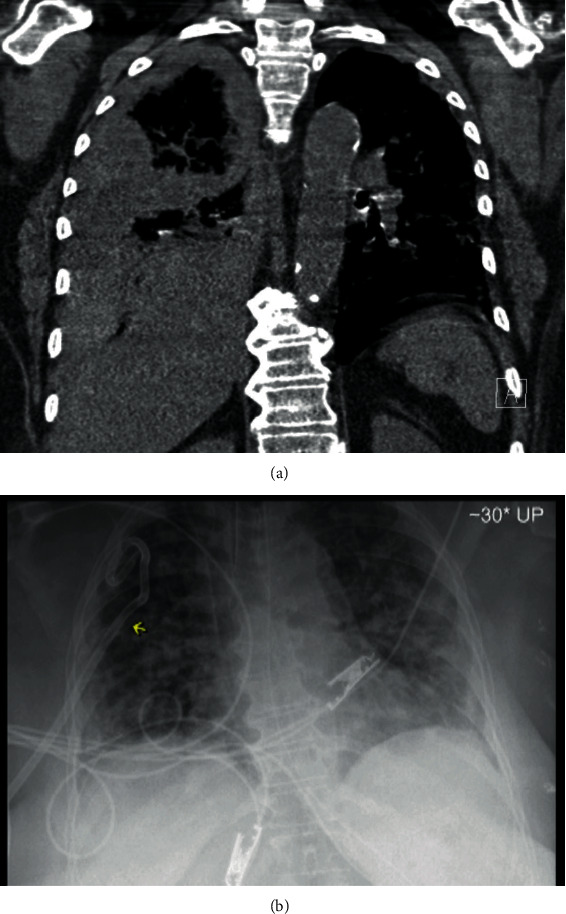
(a) Chest CT showing large right-sided hemothorax as a complication of therapeutic anticoagulation for pulmonary embolism. (b) CXR showing reexpanded lung on the right after placement of the chest tube.

A 69-year-old Hispanic morbidly obese female, nonsmoker with pertinent history of moderate persistent asthma, obstructive sleep apnea on continuous positive pressure ventilation was admitted with fever and cough and later tested positive for COVID-19. She was intubated for hypoxemia. She received azithromycin, hydroxychloroquine, and tocilizumab. On day 12, the patient suddenly started to desaturate. Her ventilatory settings were VC/AC mode, RR 35, TV 400 ml, PEEP 8 cmH20, and FiO2 of 50%. She was drawing tidal volumes of 220 ml and breathing at a rate of 35/min. CXR revealed the right-sided pneumothorax requiring immediate 14 Fr pigtail catheter placement. Subsequently, her volumes and oxygenation improved. After three days, she was extubated, but her hospital course was complicated by bilateral pulmonary emboli requiring continuous intravenous heparin infusion despite thromboprophylaxis. Unfortunately, she developed right sided hemothorax (Figure 5(a)) from the therapeutic anticoagulation and had a 28 Fr surgical chest tube and drainage (Figure 5(b)). The patient's chest tube was removed six days later, and she was discharged a few days later for short term rehab.

## 3. Discussion

Pneumothorax has been reported with many infections like *Influenza* [11], *Herpes simplex virus* [12], and *Pneumocystis pneumonia* [13]. Although acute respiratory distress syndrome (ARDS) is the primary cause of mortality in COVID-19 infection, early diagnosis and treatment of serious complications such as pneumothorax [14, 15], pneumomediastinum [16, 17], or pulmonary embolism [18] also carry utmost importance.

Incidence and an average number of days for the development of iatrogenic pneumothorax in mechanically ventilated ARDS patients are unclear. However, studies suggest that the incidence has reduced since the advent of the protective lung ventilation strategy [6]. Older studies from the preprotective lung strategy era reported that the pneumothorax mostly developed within the first couple of weeks of mechanical ventilation, and the risk decreased with time [19, 20]. In light of the current practice, pneumothorax incidence depends on the severity and duration of ARDS and ventilator mode.

Spontaneous pneumothorax has been reported in multiple case reports in COVID-19 infection [9, 21–24]. Anecdotal evidence suggests that the natural course of the COVID-19 infection predisposes to pneumothorax development [25, 26]. Even though the mechanism of the injury is not fully understood, there are several proposed theories. Like other respiratory infections related to alveolar injury, the damage from the COVID-19 infection and resultant rupture of the alveolar wall causes cystic formations within the lungs. The ischemic parenchymal damage, injury from the activation of the inflammatory processes, and fibroblast activation all precipitate fibro-myxoid exudates, promoting pulmonary cystic lesions [25, 27]. Some researchers have hypothesized that increased respiratory effort and persistent cough generate severe intrapulmonary strain, which results in the rupture of alveolar cysts [28]. Among patients requiring long-term respiratory support like in COVID-19 pneumonia, mechanical ventilation creates a persistent alveolar pressure gradient that could accelerate the cyst rupture leading to air escape of out of pulmonary tissue [29]. This air could traverse through visceral pleura and form subpleural air cysts [30]. Pneumothorax often occurs through the rupture of these subpleural air cysts. Occasionally, escaped air dissects along the perivascular and peribronchial vascular sheath into the mediastinum, retroperitoneum, and subcutaneous tissue leading to pneumomediastinum, pneumoperitoneum, or subcutaneous emphysema, respectively. Pneumothorax can also occur when mediastinal pleura rupture after the occurrence of pneumomediastinum [31, 32]. Furthermore, the probability of such devastating complications is exacerbated by the high-pressure mechanical ventilation, especially for a prolonged duration, as it triggers the formation and progression of new alveolar cysts that eventually rupture, resulting in the spread of air into various interconnected body spaces.

Clinically, pneumothorax can range from an asymptomatic to a life-threatening condition, and the management strategy depends on the degree of clinical compromise. Small pneumothorax in clinically stable patients can be managed by observation; however, larger pneumothorax with hemodynamic instability mandating active intervention to prevent catastrophic sequelae [31, 32]. Blood patch may not be useful since the patients have spontaneous coagulation abnormalities and or are on anticoagulation. If air leak is persistent despite drainage, thoracoscopy with resection of blebs and pleural scratch could be a treatment choice [33]. Potential strategies that need to be investigated include identifying individuals at high risk for pneumothorax like those with underlying parenchymal lung disease and developing a newer lung ventilation strategy pertinent to patients with COVID-19 infection to avoid volume or pressure-induced alveolar injury and subsequent complications. Several case reports have shown that barotrauma-related pneumothorax and pneumomediastinum occur in mechanically ventilated patients with COVID-19 infection. Among these with pneumothorax, tube thoracostomy, and drainage has shown satisfactory outcomes [33]. In a retrospective study by McGuinness et al., barotrauma-related complications were reported to occur in 24% of patients. This study also noted that barotrauma was an independent risk factor for death in COVID-19 infection. [34].

A recent multicenter case series from hospitals across the United Kingdom also noted pneumothorax and pneumomediastinum in patients with COVID-19 infection. They evaluated whether pneumothorax is an independent marker of poor prognosis in patients with this complication. This study also included nonintubated patients, suggesting that patients with no preexisting lung disease and who have not required positive pressure ventilation were also susceptible to pneumothorax; therefore, barotrauma alone cannot explain this association [35]. In the above case series, only two among the sixty patients with pneumothorax developed bilateral pneumothoraces sequentially. Unlike in our case series, four out of five had bilateral pneumothoraces, and three of them died despite bilateral chest tube placements. Though a small sample size, our case series suggests that pneumothorax could be a risk factor for recurrent pneumothoraces. In the case series by Martinelli, it was also observed that 28-day survival was not different among patients with COVID-19 infection even in those with pneumothorax, suggesting that pneumothorax was not associated with poor prognosis in COVID-19 pneumonia. The lung damage caused by superimposed bacterial infections likely predisposes the lung to multiple pneumothoraces, as seen in 3 out of 5 of our patients. The difference in the results observed in our case series could be explained by the small sample size, comorbidities of the patients such as four out of five patients in our study were morbidly obese, and three out of five had the underlying pulmonary disease at the time of admission.

The major limitations of our study include small sample size and the retrospective nature; therefore, we are unable to determine the incidence of pneumothorax and evaluate the differences in mortality rate and overall survival for patients with COVID-19 infection with or without barotrauma. Besides, an observational case series like ours cannot prove the causality of pneumothorax due to COVID-19 infection.

Our study results can be used to fuel future studies to assess whether pneumothorax can be used as a prognostic factor, whether it is an independent marker of mortality, especially among the critically ill patients with COVID-19 infection. Furthermore, managing patients with pneumothorax with ARDS on mechanical ventilation can be challenging if they are refractory to chest tube drainage with persistent air leak. Our study can be further developed to evaluate patients with a persistent air leak and whether the possible need for surgical intervention for pneumothoraces would provide a mortality benefit.

## 4. Conclusion

Pneumothorax can be a life-threatening complication of severe lung injury from COVID-19 pneumonia and the high ventilator setting required to manage it. We believe secondary infections also may play a role by causing further lung damage. Immediate recognition of this complication is of utmost importance, as one episode of pneumothorax could be a risk factor for recurrent pneumothorax. Though the true incidence of pneumothorax in mechanically ventilated patients with COVID-19 infection is unknown, our case series suggests that it is an appreciable number and should be kept in mind if there is a sudden change in the clinical status of an intubated patient. Continued use of already proven lung-protective ventilation strategies and interventions to prevent secondary bacterial infections would also help.

## Figures and Tables

**Table 1:** Patient characteristics.

Case	Age	Sex	Race	Comorbidities	Smoking history	Days on intubation during the event	Mode of ventilation	Ventilator settings at the time of the event (FiO2%) (PEEP)	Superimposed pneumonia	Severity of pneumothorax	Management	Outcome
1	62	M	African American	Obesity, bullous lung disease, CAD, OSA	Nonsmoker	49	PC-AC	65%, 10	Staphylococcus aureus pneumonia	Bilateral pneumothoraces	Chest tube drainage	Deceased
2	60	M	Caucasian	Obesity, HTN, OSA	Nonsmoker	14	PRVC-AC	80%, 8	Escherichia coli pneumonia	Right-sided tension pneumothorax, subsequent left-sided moderate pneumothorax	Chest tube drainage	Deceased
3	58	M	Caucasian	Obesity, HTN, MVP	Former smoker	31	VC-AC	50%, 8	N/A	Right-sided pneumothorax, left-sided tension pneumothorax	Chest tube drainage	Deceased
4	64	M	Caucasian	CAD	Former smoker	25	PC-AC	40%, 6	Staphylococcus aureus and gram-negative pneumonia	Bilateral pneumothoraces	Chest tube drainage	Survived
5	69	F	Hispanic	Obesity, asthma	Nonsmoker	12	VC-AC	50%, 8	N/A	Right-sided pneumothorax	14 Fr pigtail catheter	Survived

CAD: coronary artery disease; FiO2: fraction of inspired oxygen; HTN: hypertension; MVP: mitral valve prolapse; PC-AC: pressure control-assist control; PEEP: positive end expiratory pressure; PRVC-AC: pressure regulated volume control-assist control; OSA: obstructive sleep apnea; VC-AC: volume control-assist control.

## Data Availability

The data used to support the findings of this study are available from the corresponding author upon request.
